# The Hawaiian Freshwater Algal Database (HfwADB): a laboratory LIMS and online biodiversity resource

**DOI:** 10.1186/1472-6785-12-22

**Published:** 2012-10-25

**Authors:** Alison R Sherwood, Norman Wang, Amy L Carlile, Jessica M Neumann, Thomas K Wolfgruber, Gernot G Presting

**Affiliations:** 1 Department of Botany, University of Hawaii at Manoa, 3190 Maile Way, Honolulu, Hawaii 96822, U.S.A; 2 Department of Molecular Biosciences and Bioengineering, University of Hawaii at Manoa, 1955 East–West Rd, Honolulu, Hawaii 96822, U.S.A; 3 Present address: Department of Biology and Environmental Science, University of New Haven, 300 Boston Post Road, West Haven, Connecticut, 06516, U.S.A

**Keywords:** Algae, Biodiversity survey, Freshwater, Hawaii, Hawaiian Freshwater Algal Database, HfwADB

## Abstract

**Background:**

Biodiversity databases serve the important role of highlighting species-level diversity from defined geographical regions. Databases that are specially designed to accommodate the types of data gathered during regional surveys are valuable in allowing full data access and display to researchers not directly involved with the project, while serving as a Laboratory Information Management System (LIMS). The Hawaiian Freshwater Algal Database, or HfwADB, was modified from the Hawaiian Algal Database to showcase non-marine algal specimens collected from the Hawaiian Archipelago by accommodating the additional level of organization required for samples including multiple species.

**Description:**

The Hawaiian Freshwater Algal Database is a comprehensive and searchable database containing photographs and micrographs of samples and collection sites, geo-referenced collecting information, taxonomic data and standardized DNA sequence data. All data for individual samples are linked through unique 10-digit accession numbers (“Isolate Accession”), the first five of which correspond to the collection site (“Environmental Accession”). Users can search online for sample information by accession number, various levels of taxonomy, habitat or collection site. HfwADB is hosted at the University of Hawaii, and was made publicly accessible in October 2011. At the present time the database houses data for over 2,825 samples of non-marine algae from 1,786 collection sites from the Hawaiian Archipelago. These samples include cyanobacteria, red and green algae and diatoms, as well as lesser representation from some other algal lineages.

**Conclusions:**

HfwADB is a digital repository that acts as a Laboratory Information Management System for Hawaiian non-marine algal data. Users can interact with the repository through the web to view relevant habitat data (including geo-referenced collection locations) and download images of collection sites, specimen photographs and micrographs, and DNA sequences. It is publicly available at http://algae.manoa.hawaii.edu/hfwadb/.

## Background

Biodiversity surveys of poorly understood groups of organisms, including algae, are especially critical in isolated geographical regions that may harbour unique diversity. Results of such surveys are of interest to those who specialize in study of those groups of organisms, as well as ecologists, conservation and restoration biologists and natural resource managers. The Hawaiian marine algae, although not completely characterized, have received far more attention through systematic study and large-scale biodiversity inventory efforts [1-3] than have the non-marine algae that occupy such diverse habitats as streams, ponds, lakes, ditches, agricultural fields, cave and lava tube walls, wet walls, terrestrial and subaerial surfaces, and high elevation bogs. The Hawaiian Freshwater Algae Biodiversity Survey was established in 2009 to characterize all of the non-marine algae of the Main Hawaiian Islands through analysis of samples collected throughout the main islands of the archipelago over a three-year period. Collection sites are documented with photographs and GPS coordinates. Algal samples are characterized depending on the conventions for that particular lineage, usually including microscopic and molecular characterization, and sometimes culturing studies. Vouchers of all collections are retained in the Sherwood Laboratory at the University of Hawaii, typically preserved in glutaraldehyde on glass microscope slides. These vouchers will ultimately be deposited in the Bernice Pauahi Bishop Museum (BISH) in Honolulu. DNA extractions were performed on all samples that were sufficiently large and epiphyte-free, and DNA barcode and phylogenetic markers were amplified and sequenced according to the published conventions for each lineage of algae. These DNA sequence data comprise the first molecular reference data set for Hawaiian non-marine algae, and are a critical resource for comparing potential alien or invasive species to the state, as well as for evolutionary studies.

Access to these kinds of specimen-based biodiversity data has traditionally been cumbersome, often relying upon loans from individual researchers, herbaria or museums housing the specimens. Dissemination of our results has been facilitated by the development of the custom-designed Hawaiian Freshwater Algal Database (HfwADB), an Internet (web)-accessible repository of specimen data. HfwADB is a modified version of the earlier Hawaiian Algal Database (HADB) developed for the Hawaiian Rhodophyta Biodiversity Survey [4], and can accommodate additional layers of information associated with samples containing multiple species. This modification proved to be necessary since most non-marine algal samples are collected from the field in the form of mixed-species assemblages (e.g., a scraping of an algal biofilm containing a number of algal species) rather than as algal individuals (i.e., most collections from the marine environment collected under the previous Hawaiian Rhodophyta Biodiversity Survey). HfwADB serves as both a project-based internal Laboratory Information Management System (LIMS) and a public portal for accessing the biodiversity survey data associated with the Hawaiian Freshwater Algal Biodiversity Survey.

## HfwADB construction and content

HfwADB was built using MySQL and PHP to store and display the various types of data collected during our biodiversity survey. The web interface is programmed in PHP, using Smarty Template Engine (http://www.smarty.net/) to separate content from presentation. The web interface makes use of Cascading Style Sheets and is designed to be usable in browsers that do not support graphics; however, the recommended browser is Mozilla Firefox. There are two modes of interface – user and administrator. The user interface is for browsing only. The administrator interface enables additional functions for logged-in users with adequate privileges. The data input pages and the administrator mode console are double checked for sufficient privilege when executing the functions for any data alteration operations. Various table columns have additional indices constructed to optimize complex join query speed. The database schema has been normalized into third normal form, to avoid storage of data with logical inconsistencies. Images are stored within the database as BLOBs (Binary Large Objects) to streamline resizing, watermarking and backup: multiple versions of photos are not stored on disk; instead, they are generated (resized and watermarked) on-the-fly upon request (without noticeable lag).

**Figure 1:**
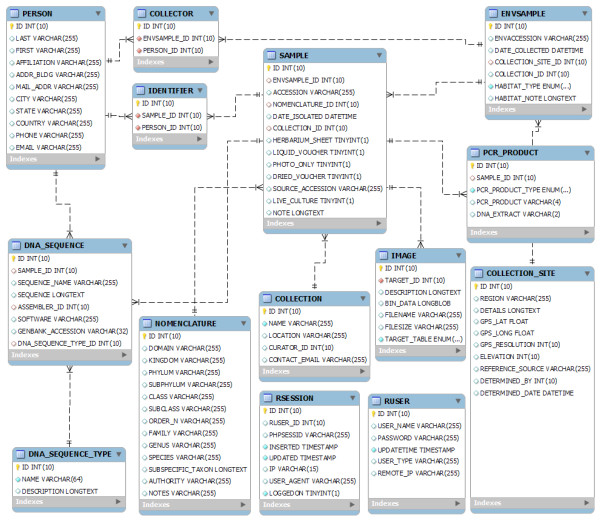
**The database schema. **Database schema: organization of the Hawaiian Freshwater Algal Database and relationships between the 14 tables.

HfwADB runs on a LAMP system: Linux (CentOS), Apache, MySQL, and PHP. The MySQL database is segmented into fourteen tables: COLLECTION, COLLECTION_SITE, COLLECTOR, DNA_SEQUENCE, DNA_SEQUENCE_TYPE, ENVSAMPLE, IDENTIFIER, IMAGE, NOMENCLATURE, PCR_PRODUCT, PERSON, RSESSION, RUSER and SAMPLE (Figure 1). Web traffic is tracked at three levels: remote scripts, local scripts and server logs. Remote cross-site scripts from Google Analytics monitor general user traffic; local scripts via PHP sessions monitor user-specific traffic and Apache server logs provide overall web server access statistics.

The HfwADB server is housed in a cooled, dehumidified server room at 20°C. The server utilizes an Intel Core 2 Duo 2.4GHz 6600 processor and 6GB of DDR2 RAM. The rack mount server case contains eight hot swappable hard disk bays and is housed in a water resistant server rack. Surge protection and an uninterruptible power supply are provided. A second power supply is available in case of failure. The server is designed to make several copies of the database to safeguard against catastrophic hardware failure or hacking. The data is both mirrored in real-time using software RAID1 and backed up incrementally onto the hardware RAID 6 disk array.

The hardware RAID 6 will remain operational with up to two disk failures, and will additionally automatically attempt to rebuild the array the moment one hard drive fails using a single global hot-spare. The incremental backup is performed by custom rsync shell scripts where unchanged files are copied as hard links (to save storage space) and only the delta of changed files is copied to reduce time to transfer files. The scripts are automatically launched by a set of cron jobs every six hours (saved up to a day), every day (saved up to a week), every week (saved up to four weeks), and every month (saved up to 12 months).

## Utility and discussion

Data generated from natural history collections, especially for freshwater algae, are often available only in the form of static publications at the conclusion of a study. Our goal was to produce a dynamic, open-access database that illustrates the progress of our biodiversity survey to encourage constructive feedback and promote collaboration. The database design focuses on the two levels of organization most important to this project – that of the collection site (Environmental Accession) and the individual alga (Isolate Accession). Currently, HfwADB is populated with data for 1,786 Environmental Accessions (from samples spanning six islands), 2,825 Isolate Accessions, and hosts a total of 4,985 images. Twenty-three people were involved in the collecting of samples for the survey, and seven researchers performed identifications on the material collected. HfwADB contains 895 DNA sequences: 298 nuclear or cyanobacterial 16S, 544 plastid and 53 mitochondrial. The Isolate Accessions in HfwADB represent diverse algal lineages found in non-marine habitats worldwide, including cyanobacteria (80 accessions representing 26 genera), green (1,411 accessions representing 54 genera) and red algae (155 accessions representing six genera), diatoms (1,085 accessions representing 55 genera), yellow-green algae (45 accessions representing two genera) and euglenoids (19 accessions representing two genera), along with some isolates that could not be identified or were isolated as contaminant DNA sequences from a culture (30).

One of the most valuable products of this biodiversity survey is the DNA sequence data that have been generated for as many of the Isolate Accessions as possible. These data allow algae from the Hawaiian Islands to be compared with collections from other parts of the world. Our approach has been to focus on short, standardized DNA sequences that can be compared using a DNA barcoding approach [5] while complementing these with sequences of longer markers that are commonly used to elucidate phylogenetic patterns in particular algal lineages.

Construction of HfwADB was funded under a federal grant from the U.S. National Science Foundation to document and characterize the non-marine algae of the Hawaiian Islands. Design and construction of HfwADB was one of the first goals of the project and was largely completed in 2010. As of this writing (October 2012), this particular grant is in its final year, but data are still being added to the database, and we foresee using, maintaining and supporting this resource for many years to come.

### Database use

Upon accessing the database website (http://algae.manoa.hawaii.edu/hfwadb/), users begin with a simple search interface that includes multiple options (various levels of taxonomy, island or keywords associated with a collection site, habitat type [stream, wet wall, reservoir, lake, bog, taro field, ditch, terrestrial, estuary, or “other”] or accession number [5-digit Environmental Accession or 10-digit Isolate Accession with a hyphen separating the first and last five digits]). Multiple accessions can be searched by entering accession numbers separated by a space. Simple search instructions and background information on the database can be found by hovering the mouse pointer over sections on the main page. A Basic Local Alignment Search Tool (BLAST) function [6] is linked on the main page whereby users can enter a DNA sequence for one of the markers included in the database and BLAST hits and alignment comparisons to the sequence content of HfwADB will be returned.

**Figure 2:**
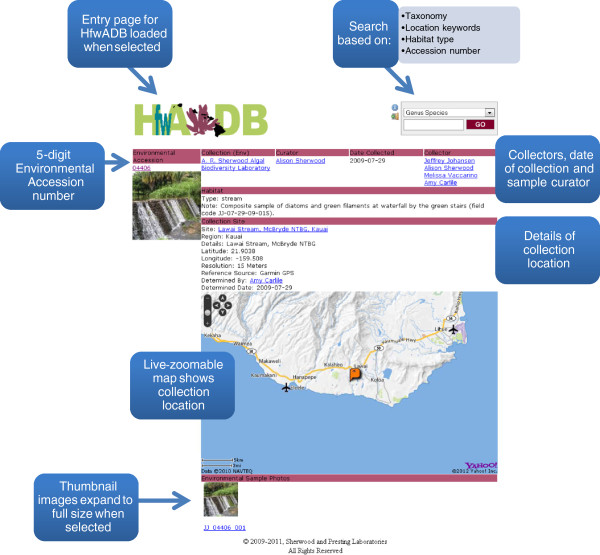
**Example database environmental accession page. **Screenshot of an Environmental Accession page illustrating the types of data stored for collection sites.

**Figure 3:**
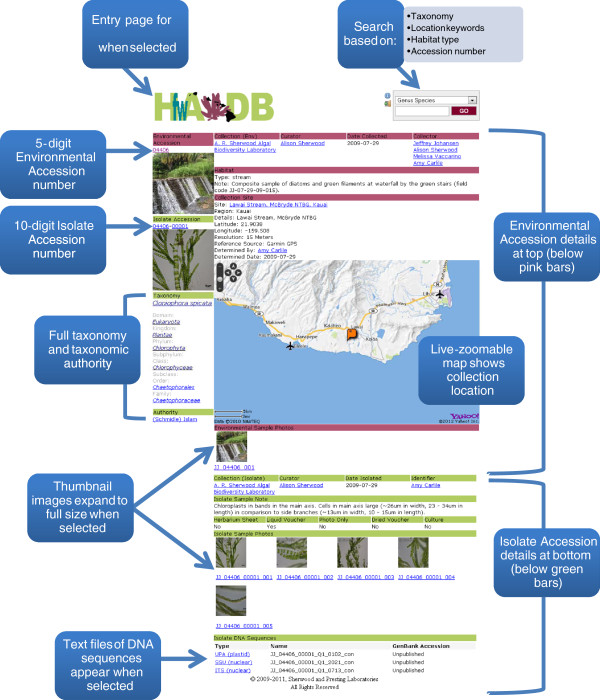
**Example database isolate accession page. **Screenshot of an Isolate Accession page illustrating the types of data stored for individually identified algae.

The results page returned from any query includes eight columns of information: Environmental Accession, Isolate Accession, Organism (genus and species), Region (island), Details (collection location), Collected (date), Isolated (date) and Sequences (total number of DNA sequences). Users may select either the Environmental Accession to display information about the collecting location, or the Isolate Accession to display the full range of data available for a particular identification. The Environmental Accession page (Figure 2) includes details of when and where the sample was collected, the geo-referenced collection site displayed using the Yahoo! Maps service API embedded within the website, an indication of habitat type and any field notes that were taken, and at least one photograph of the collection site (which can be viewed at higher resolution by selecting it). The Isolate Accession page (Figure 3) includes both Environmental and Isolate Accession details pertaining to that particular sample. Environmental Accession data are displayed at the top of the page (under pink heading bars), in much the same way as for an Environmental Accession page, while Isolate Accession data are displayed toward the bottom of the page (under green heading bars). The full taxonomy and taxonomic authority of the identified alga are displayed on the left-hand side under the thumbnail images of the Environmental and Isolate Accessions. Details of where the alga is stored [Collection (Isolate)] along with the Curator of the collection, the Identifier of the sample and the date it was identified (Date Isolated) are shown under the Environmental Accession data. Notes that may be helpful to others in identifying the alga are included under Isolate Sample Note, followed by information on the type of voucher (Herbarium Sheet, Liquid Voucher, Photo Only, Dried Voucher and/or Culture) and thumbnail images of key characters of the taxon with compound light or dissecting microscopy (which are displayed at higher resolution and with scale bars when selected). At the bottom of the panel is a list of DNA sequences (Isolate DNA Sequences) associated with that alga, which may include one or more loci from the nucleus (or principal chromosome, as in the cyanobacteria) (ITS = Internal Transcribed Spacer region(s) [7], LSU = nuclear ribosomal large subunit gene [8], SSU = nuclear ribosomal small subunit gene [9], 16S = gene encoding the ribosomal small subunit in prokaryotes including cyanobacteria [10]), plastid (*rbcL* = gene encoding the large subunit of the Rubisco enzyme [11], *tuf*A = gene encoding elongation factor Tu [12], UPA = Universal Plastid Amplicon [13,14], ) or mitochondrion (COI = 5^′^ cytochrome oxidase subunit I gene [15]).

## Conclusions

Given the disparate forms of data associated with each specimen in large inventories such as the Hawaiian Freshwater Algal Biodiversity Survey, use of a project database has clear value to those responsible for collecting, analyzing and curating the data. Thus, HfwADB has been a critical component of project success, serving as a LIMS for the Sherwood Laboratory. Researchers studying non-marine algae in other regions are able to easily search for taxa of interest through HfwADB and may contact the authors if interested in using these samples for comparative analyses. Beyond this, HfwADB will be a key resource for biologists and resource managers working in the tropical Pacific, allowing others to compare details of collecting sites and the key characters for identification of specimens to their own samples. No other such resource for non-marine algae currently exists for any tropical region of the world.

## Availability and requirements

The Hawaiian Freshwater Algal Database is publicly available at http://algae.manoa.hawaii.edu/hfwadb/. The template MySQL database and PHP scripts are available upon request from the authors.

## Competing interests

The authors declare that they have no competing interests.

## Authors’ contributions

AS wrote the manuscript. GP advised implementation of the MySQL database, PHP scripts, interface design, and compiled database statistics. TW compiled database statistics. AS, AC, JN collected specimen data and submitted them to the database. AS and GP served as database curators and project advisors and obtained the funding for the project. All authors checked the accuracy of the database and web interface, and read and approved the final manuscript.
